# Development and Characterization of LLDPE Blends with Different UHMWPE Concentrations Obtained by Hot Pressing

**DOI:** 10.3390/polym14183723

**Published:** 2022-09-06

**Authors:** Pollyana Melo Cardoso, Marcelo Massayoshi Ueki, Josiane Dantas Viana Barbosa, Willams Teles Barbosa, Benjamin Lazarus, Joyce Batista Azevedo

**Affiliations:** 1 Department of Materials, University Center Senai Cimatec, Salvador 41650-010, Bahia, Brazil; 2 Graduate Program in Materials Science and Engineering—P2CEM, Federal University of Sergipe (UFS), Aracaju 49100-000, Sergipe, Brazil; 3 Pós-Graduate Program PPGGETEC, University Center Senai Cimatec, Salvador 41650-010, Bahia, Brazil; 4 Materials Science and Engineering Program, University of California San Diego, San Diego, CA 92093, USA; 5 Institute of Science, Technology and Innovation, Federal University of Bahia, Salvador 42809-000, Bahia, Brazil

**Keywords:** blends, LLDPE, UHMWPE, hot pressing

## Abstract

To modify its characteristics, expand its applicability, and, in some cases, its processability, new blends using ultra-high-molecular-weight polyethylene (UHMWPE) have been developed. In this study, three different formulations of linear low-density polyethylene (LLDPE) and UHMWPE blends were prepared with 15, 30, and 45% (% *w*/*w*) UHMWPE in the LLDPE matrix. All mixtures were prepared by hot pressing and were immersed in water for one hour afterwards at a controlled temperature of 90 °C to relieve the internal stresses that developed during the forming process. The thermal characterization showed that the blends showed endothermic peaks with different melting temperatures, which may be the result of co-crystallization without mixing between the polymers during the forming process. The mechanical characteristics presented are typical of a ductile material, but with the increase in the percentage of UHMWPE, there was a decrease in the ductility of the blends, as the elongation at rupture of the blends was higher than that of the pure components. The morphologies observed by SEM indicate that there were two phases in the blends. This is the result of the system’s immiscibility due to the mode of preparation of the blends, wherein the two polymers may not have mixed intimately, confirming the results found with the thermal analyses.

## 1. Introduction

Interest in polymer blends has been steadily increasing over the past few years [1]. Mixing two or more matrices can create new polymers with improved properties, different from their constituents, and can overcome traditional problems that arise when synthesizing new polymer species [2]. Generally this strategy is more economically viable and can develop a wide range of materials with better functionality and formability from the modification of their composition [3]. 

The formation of blends from the mixture of two polyethylenes is being increasingly evaluated for several applications. The use of UHMWPE is increasing mainly due to the search for the modification of its characteristics and, consequently, the expansion of the applicability of this material, or in some cases, just to facilitate its processing [4,5,6,7,8]. 

UHMWPE is a thermoplastic, semicrystalline, linear homopolymer composed of hydrogen and carbon [9]. It belongs to an emerging class of high-performance specialty polymers with a unique set of properties and applications [10]. UHMWPE has considerable similarities to other polyethylenes, but its extremely high molar mass and extensive give rise to its unique properties [11]. High-density polyethylene (HDPE) resembles UHMWPE in terms of molecular structure, crystal melting temperature, permeability, and chemical inertness. However, UHMWPE has exceptional low-temperature impact strength, high abrasion resistance, and excellent resistance to the development of stress cracking due to the low coefficient of friction, as well as high fatigue resistance, excellent self-lubrication, and both thermal and acoustic insulating properties [12]. A major limitation of this material, however, is its processability. Even above its melting temperature, UHMWPE still has high viscosity and cannot be formed by the conventional techniques used for other thermoplastics, with the exception of compression molding and RAM extrusion [13]. 

The molecular chains of UHMWPE have high random entanglement density, which makes their thermal motion response sluggish, resulting in low molecular chain mobility and limited compatibility with other polymers. The addition of LLDPE, a material with a similar structure to HDPE and a high fluidity index, can improve processing by reducing the viscosity of the melt. Furthermore, the structural similarity between the polymers can lead to the formation of compatible and/or miscible systems [7,14]. 

LLDPE is a copolymer of ethylene with an α-olefin (propene, 1-butene, 1-hexene, or 1-octene). This polymer consists of uniformly sized, short branched chains produced through the copolymerization process. The crystallinity of this polyethylene is between 30–40%; it has good mechanical strength, absorbs little humidity and is inexpensive and easy to process [15,16]. 

The performance of polymer blends depends on the properties of the polymer components, as well as on how they are arranged in space. The spatial arrangement is controlled by the thermodynamics and morphology imposed by the phases. The properties of a polymer blend depend on the constituent polymers, on the type of morphology formed during processing, and in some cases, on the agents used to favor phase compatibilization [17]. 

Therefore, the objective of this work was to study blends of LLDPE and UHMWPE obtained by pressing and thus evaluate the mechanical, thermal, and morphological properties. To obtain the blends, hot pressing by compression was used. This forming method creates plastic parts with low cost and high productivity and suits the processing conditions of UHMWPE [18,19]. 

## 2. Materials and Methods

### 2.1. Materials

In this work, LLDPE (ML 3601U) with a hexene comonomer, flow index of 3.3 g/10 min, and density of 0.939 g/cm^3^ was used. UHMWPE (UTEC 6540), in powder form, with a density of 0.925 g/cm^3^, was added to the LLDPE. Both polyethylenes were supplied by Braskem S.A. (Camaçari, Brazil).

### 2.2. Methods

Initially the LLDPE was micronized in a micronizer, model TM460, for polymers (Tritumaq, São Paulo, Brazil), with a rotation speed of 1750 rpm, sieves of 18 mesh, and a micronization chamber temperature at 60 °C. UHMWPE was used in powder form, as supplied by the manufacturer. The particle size distribution of the polymers was determined by sieving according to ASTM D 6913-04 [20]. 

Blends were produced at proportions of 15, 30, and 45% (% *w*/*w*) of UHMWPE in the LLDPE matrix, named respectively 15 UHMWPE, 30 UHMWPE, and 45 UHMWPE. Initially, the polymers, both in micronized powder form, were premixed manually in a plastic bag by vigorous shaking for approximately 5 min. After manually mixing the polymers, plates were obtained by hot pressing. For this, an advanced press was used (Advanced do Brazil, Novo Hamburgo, Brazil), with a plateau heated by electrical resistance, pressure of 60 kgf/cm^2^, and an opening speed of 200 mm/s. A square mold made of 316 stainless steel, measuring 195 × 195 × 4 mm, was used. To relieve the stresses from the forming process, the specimens were immersed in water for one hour at a controlled temperature of 90 °C. There was warpage in the plates after demolding and an insignificant variation in thickness. 

The time and temperature parameters used in the forming process were established according to the thermal properties of LLDPE and previous experimental analyses. Therefore, the samples were heated to 160 °C for 30 min and thus presented suitable forming conditions for samples in the form of plates. Thermal characterization was performed in a TA Instruments DSC model Q3 (TA Instruments, New Castle, USA), with a heating rate of 10 °C/min in a nitrogen atmosphere, over a temperature range of 23 to 200 °C, with two heating and one cooling runs. The first heating aimed to reveal the effect of the thermal history of the material during the forming of the plates on the thermal properties of the blends and the second heating cycle, after controlled cooling, to study the effect of the composition of the blends on the thermal properties of the same. To calculate the degree of crystallinity (Xc), Equation (1) was used, wherein the enthalpy of the 100% crystalline LLDPE used was 292 J/g and the enthalpy of the 100% crystalline UHMWPE used was 293 J/g [21], according to the rule of mixtures. With this analysis, the melting temperature (Tm), melting enthalpy (ΔHm), and crystallization temperature (Tc) were determined.

(1) $$\mathrm{Xc}=\frac{\Delta \mathrm{Hm}\left(\mathrm{sample}\right)}{\Delta \mathrm{Hm}\left(\mathrm{Polyethylene}\;100\%\;\mathrm{crystalline}\right)}$$

The mechanical properties under tension were obtained in an EMIC-brand equipment model DL 200 (EMIC, São Paulo, Brazil) following ISO 527-1: 2019 standard [22] type 5A with strain gauge. The modulus of elasticity (MPa), tensile strength (MPa), and strain at rupture (%) of each formulation were obtained by averaging the results of five samples. For the modulus, it was obtained through the software of the equipment that measures the deformation of the material through a strain gauge. For the test, a displacement rate of 50 mm/min was used. During sample fabrication, temperature maps were created of the plates obtained by pressing, in order to identify the hottest areas and areas with less temperature dispersion. Using these maps, specimens were extracted from regions with the most uniform temperature to ensure more homogeneous properties. To map the press temperatures, a FLIR T300 camera-type thermal imager (Instrutemp, São Paulo, Brazil) was used, which can measure temperatures in the range of −20 to 650 °C and has a thermal sensitivity of 0.05 °C. After mapping, it was decided to remove the specimens from the central region of the mold because it presented the least temperature variation. 

X-ray diffractograms were obtained in a Shimadzu XRD 6000 diffractometer (Shimadzu Corporation, Kyoto, Japan) using CuKα radiation with wavelength λ = 1.5406 Å and a monochromator with a 2θ scanning angular range of 8–32°. The measurements were performed at room temperature in continuous scanning mode, with an angular step of 0.02° and a counting time of 1.20 s. The voltage and current used in the analyses were 30 KV and 30 mA, respectively. 

The morphology of the blends was investigated by scanning electron microscopy (SEM) using a JEOL microscope, model Carry Scopy JSM-6510LV (JEOL Ltd., Tokyo, Japan), with an accelerating voltage of 20 kV. The fracture surface from specimens after mechanical testing was analyzed. The samples were coated with gold using an evaporation metallization equipment, model DESK V (Denton Vacuum, NJ, USA).

## 3. Results and Discussion

**Figure 1:**
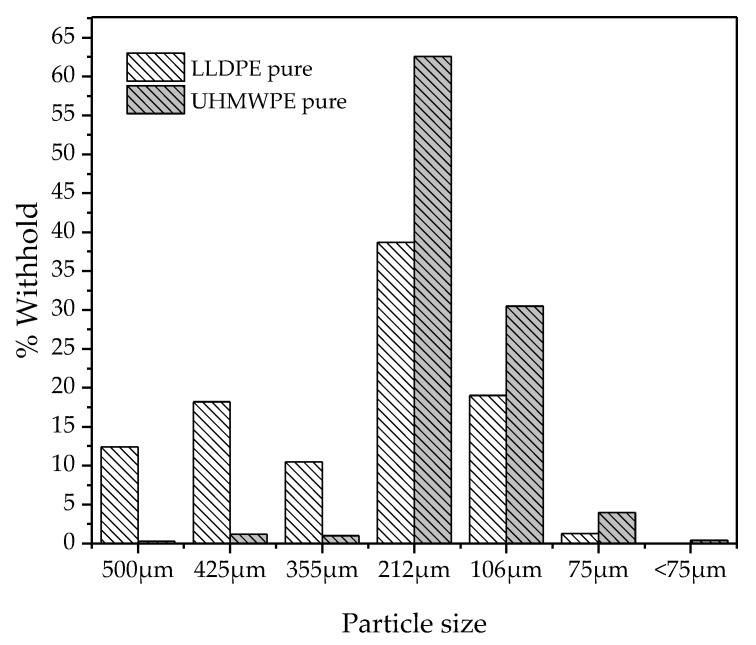
Particle size distribution of LLDPE and UHMWPE.

The particle size distribution found for LLDPE and UHMWPE in powder form was between 106 and 212 µm (Figure 1). 

**Figure 2:**
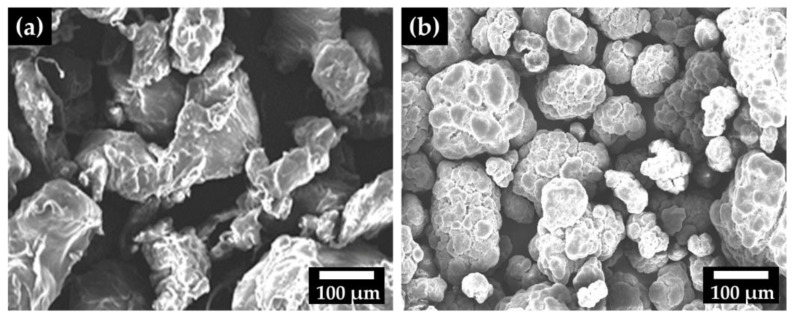
SEM images of pure polymer powders: (**a**) LLDPE, (**b**) UHMWPE.

After micronization, LLDPE contained deformed particles, different from UHMWPE, which had aggregates of particles with microvoids linked by fibrils (Figure 2). When UHMWPE is subjected to compaction and sintering, the molecular free space is reduced, and pores and weak connections are formed between the particles [23,24,25,26].

**Figure 3:**
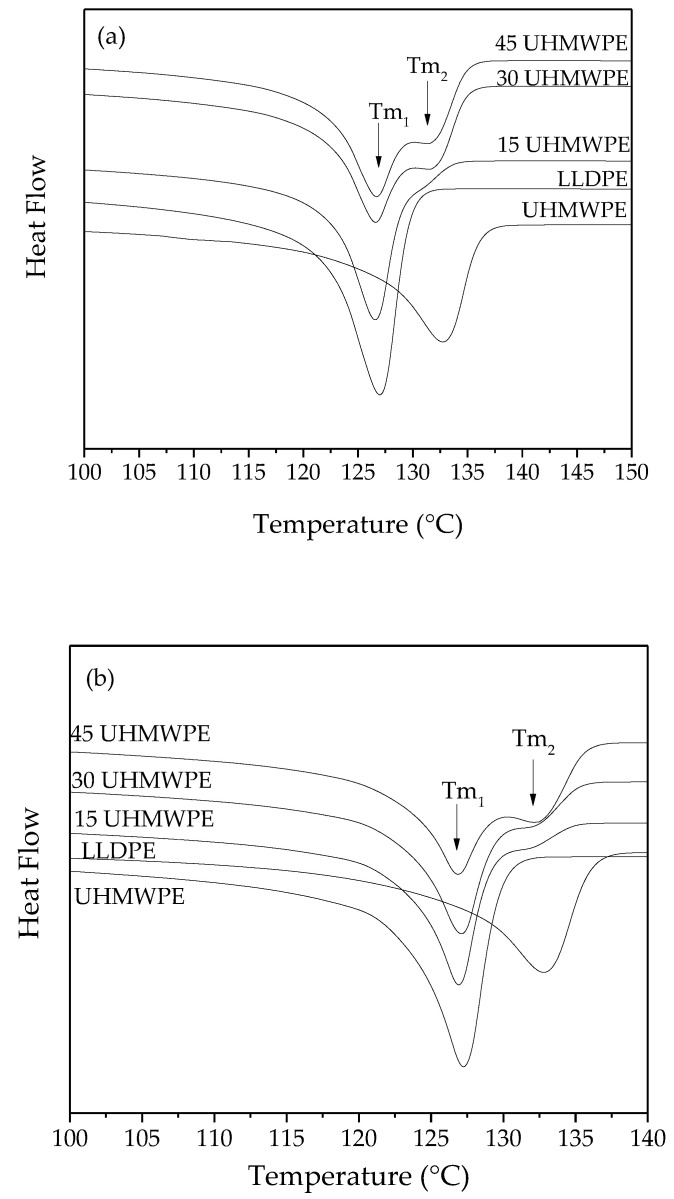

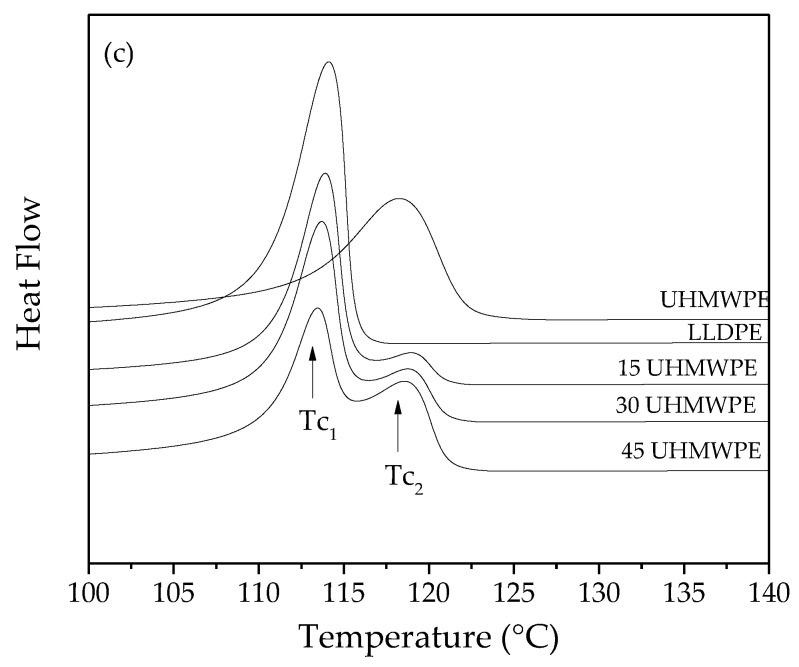
DSC curves of LLDPE, UHMWPE, and the blends: (**a**) first heating, (**b**) second heating, and (**c**) cooling.

Figure 3 shows the thermograms of LLDPE, UHMWPE, and the blends after hot pressing for the (a) first heating, (b) second heating, and (c) cooling stages. The thermograms show the melting (1st and 2nd heating) and crystallization (cooling) behavior of the blends, as well as those of pure LLDPE and UHMWPE. The heating thermograms (1st and 2nd heating) of the blends are characterized by two peaks at the melting temperature (Tm_1_ and Tm_2_), wherein Tm_1_ is attributed to the melting of LLDPE and Tm_2_ to the melting of UHMWPE (Figure 3a,b). On cooling, it is also possible to identify two crystallization peaks associated with LLDPE (Tc_1_) and UHMWPE (Tc_2_) (Figure 3c).

Table 1 and Table 2 present the values of the thermal properties obtained and calculated with the DSC thermal analysis data of the blends and the pure polymers, LLDPE and UHMWPE. 

For LLDPE and UHMWPE, melting is observed at approximately 127 °C and 132 °C, respectively. The difference in melt temperature is due to variations in chain structure between the two types of polymers. LLDPE has short branches randomly distributed along the main chain. This leads to the formation of a crystalline phase with a larger number of defects and smaller lamellae size (crystallites). Meanwhile, UHMWPE has a long linear structure, which facilitates the formation of more perfect crystals, resulting in a shift in the melting temperature peak to higher temperatures when compared to UHMWPE [27]. In the blends analyzed here, the same endothermic peaks associated with the melting of the LLDPE crystalline phase are verified, at around 127 °C, and another one associated with the UHMWPE crystalline phase is observed at approximately 132 °C. The fact that the blends show endothermic peaks at different temperatures can be explained by considering that no co-crystallization has occurred. This phenomenon may come from a non-homogeneous mixture resulting from, in the case of this study, manual mixing and hot pressing. Mixing conditions and mixing technique can have direct effects on co-crystallization and miscibility in polyethylene blends, as observed by Chen et al. [27] and Sweed, M. [28].

No significant variation of the LLDPE melt peak was identified when UHMWPE was added, showing that there is no diffusion of LLDPE chains into the UHMWPE [27]. Gai and Zuo [29] studied normal-molecular-weight-polymer (NMWP)/UHMWPE blends and demonstrated that metastable state was found to be advisable for both viscosity reduction and mechanical property improvement of the UHMWPE/NMWP blends. For Gai and Zuo [29], a normal-molecular-weight polymer means polymers such as LLDPE, HDPE, PP, and PLA. The method used for the production of the blends in our work was the hot pressing of powders instead of mixing in the melted state, may have reduced the ability to form a intimate mixture between the polymers, and maintained the phases of each constituent.

During the cooling stage, two exothermic events were observed for the blends; the first one occurred around 114 °C and was to the maximum crystallization rate (Tc) of LLDPE, while the second event occurred around 118 °C and was the Tc of UHMWPE. With the addition of 15% UHMWPE, a decrease in crystallinity was observed, but with the increasing UHMWPE content, a small increase in crystallinity to 56.3% (30 UHMWPE) and 57.5% (45 UHMWPE), compared to LLDPE and UHMWPE, 52.9% and 52.6%, respectively, was also noted (Table 2). This behavior can be attributed to the mixing conditions. The degree of crystallinity of the samples was higher in the second heating due to the favoring of the mixture and co-crystallization. Another relevant factor is the relationship with the heating rate; in the DSC the rate is lower than that applied in the press, thus favoring the molecular rearrangement.

**Figure 4:**
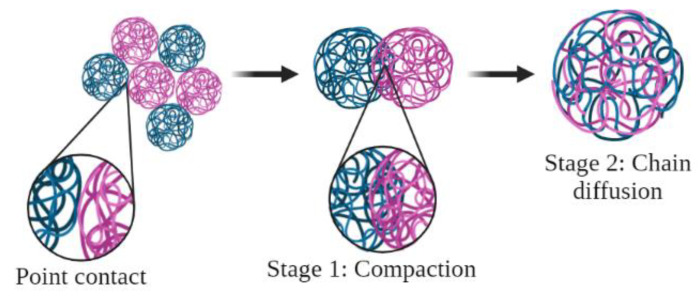
Schematic diagram of the two stages in press molding, adapted from [32].

Gao and Mackely [30] reported that the homogenization of polymers during molding can be divided into two stages (Figure 4): Stage 1, the compaction of powder and the removal of voids (Type 1 fusion defects), and Stage 2, the randomization of molecular conformations at particle boundaries by self-diffusion and the removal of memory from interfaces (Type 2 fusion defects). According to Wu et al. [31], the self-diffusion process (2nd stage) along the entire molecular length is much slower in UHMWPE. The press molding process is complete when all of the molecules are fully randomized, and all memory of the interfaces, in terms of molecular conformation, is lost. The practical problem is that both steps occur very slowly in UHMWPE. This phenomenon may explain the characteristics observed during the formation of the LLDPE/UHMWPE blends.

**Figure 5:**
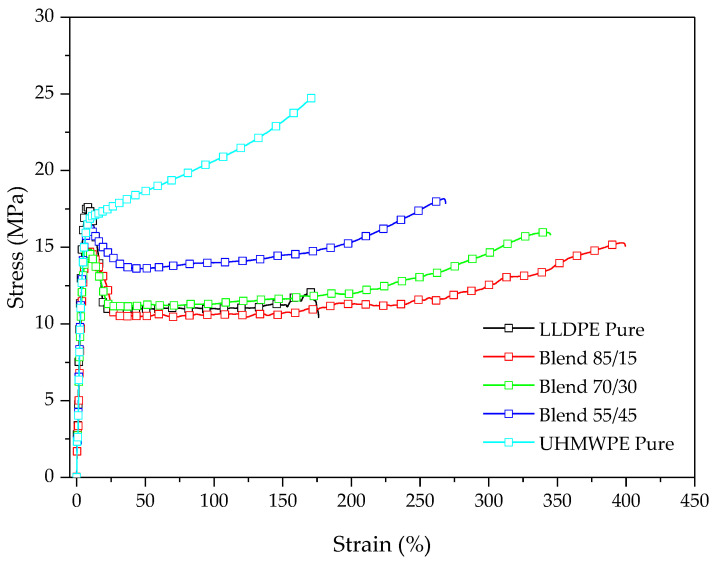
Typical stress–strain curve of LLDPE, UHMWPE, and the blends.

Figure 5 shows the typical stress–strain curves for LLDPE, UHMWPE, and the blends. Table 3 presents the data of the mechanical properties found for the blends in tensile testing. 

With increasing UHMWPE content, there is a decrease in the ductility of the blends; however, the strain at rupture (Ɛr (%)) of the blends was higher than that of the pure components, LLDPE and UHMWPE (Table 3). It is observed that an increase in tensile strength occurs with the addition of UHMWPE compared to LLDPE, which may be the result of greater entanglement and consequently increased cross-linking, when UHMWPE is added to the system, which improves the tensile capacity [32].

It is also noted the elongation at rupture of the blends is higher than that of the pure components but gradually decreases with increasing concentration of UHMWPE. Heng et al. [33] identified this same trend for LDPE/UHMWPE systems, with decreased tensile strength and elongation at break relative to the pure components. This decrease in elongation at rupture can be attributed to the wide size distribution of the dispersed phase, evidenced by the morphology of the blends and the different crystallization rates between LLDPE and UHMWPE [25,33].

With increasing UHMWPE content in the system, there is a decrease in the modulus of elasticity relative to pure LLDPE. These trends of invariance in the modulus of elasticity of the blends may be related to the dispersion of UHMWPE and the insufficient coating of LLDPE on the UHMWPE particles [25].

**Figure 6:**
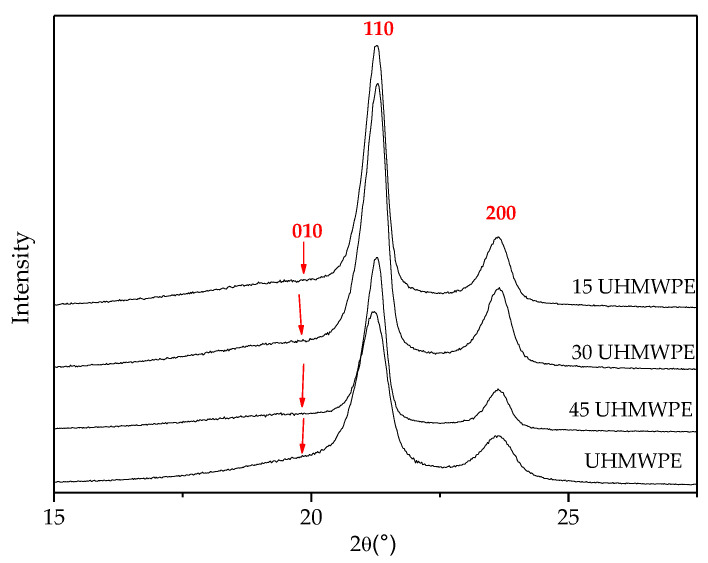
X-ray diffractograms of UHMWPE and the blends.

Figure 6 shows the X-ray diffractograms for the blends and for UHMWPE. For all of the samples, two intense peaks were identified at 21.4° (2θ) and 23.7° (2θ). This implies that the mixing of the two crystalline phases of polyethylenes with molecular-weight differences does not lead to significant changes in the alignment of the crystal planes [33]. Furthermore, there is a significant decrease in peak intensity at 21.4° (2θ) and 23.7° (2θ) The corresponding crystal plane diffraction (110) and (200) verifies the gradual increase in the amount of UHMWPE in the blends [34]. 

The X-ray diffractogram of UHMWPE shows a weak reflection very close to the (110) peak (amorphous halo). 

**Figure 7:**
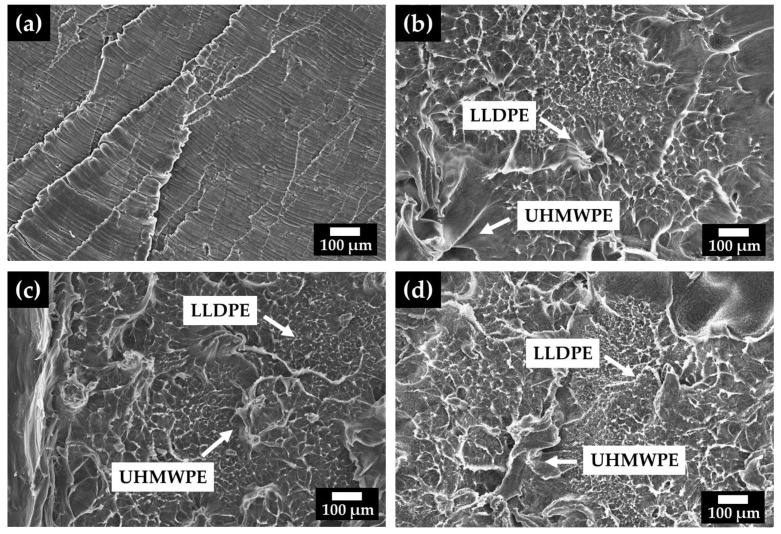
SEM micrographs of the UHMWPE and the blends. (**a**) UHMWPE, (**b**) 15 UHMWPE, (**c**) 30 UHMWPE, and (**d**) 45 UHMWPE.

Figure 7 shows the morphology of the fracture surface of UHMWPE and the blends. For all of the blends, the fibrillation aspect that characterizes a rupture surface and is typical of LLDEP was identified [32]. The types of ruptures can be divided into three different categories. Phase (i): cracking without apparent defects (appearance of voids between the fibrils), Phase (ii): the appearance of cracks (with the rupture of the fibrils), and Phase (iii): the propagation of cracks promoting their rupture [35].

**Figure 8:**
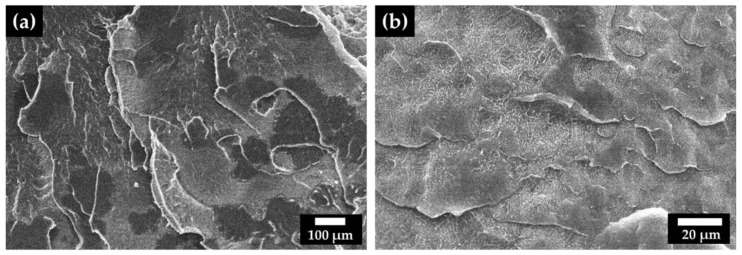
SEM micrographs of the cryogenic fracture surface of the blends: (**a**) 30 UHMWPE and (**b**) 45 UHMWPE.

To better identify the distinction between the phases, microscopy analysis of the fracture surface obtained by cryogenic fracturing was performed (Figure 8). For the blends 30 UHMWPE (Figure 8a) and 45 UHMWPE (Figure 8b), a dense network structure was identified, with grain boundaries that could be related to the lack of mixing between the phases (LLDPE and UHMWPE). The presence of two phases for the blends may characterize the immiscibility of the system or the solid-state preparation mode of the blends, wheren the two polymers may not have mixed homogeneously, corroborating the results found with the thermal analyses (Table 1 and Table 2). Chen et al. [35] showed that the LLDPE/UHMWPE system has partial microphase separation and that the phase separation depends directly on the LLDPE content. During processing, the molten LLDPE may have penetrated into the voids between the particles of the UHMWPE powder (Figure 2b). Thus, LLDPE acts as an adhesive between the particles, due to its miscibility in the melt state of UHMWPE and LLDPE. This adhesion mechanism has been analyzed by several authors [23,25,36].

For the LLDPE/UHMWPE system, immiscibility can occur, since there is no mechanical mixing in the melted state, only a pressing of powders, so there is non-homogeneous mixing of the two materials. Boscoletto et al. [36] reported that, for the HDPE/UHMWPE system, the UHMWPE particles are composed of a large number of small units, which contain microvoids between them and promote wettability within the material. This was also observed in the analyzed LLDPE/UHMWPE matrix. Therefore, the incorporation of UHMWPE in the blends with other polyethylenes plays an important role, due to both the high-molecular-weight chain tails dissolving in the continuous phase (LLDPE), and since they remain as suspended phase particles in the blend.

**Figure 9:**
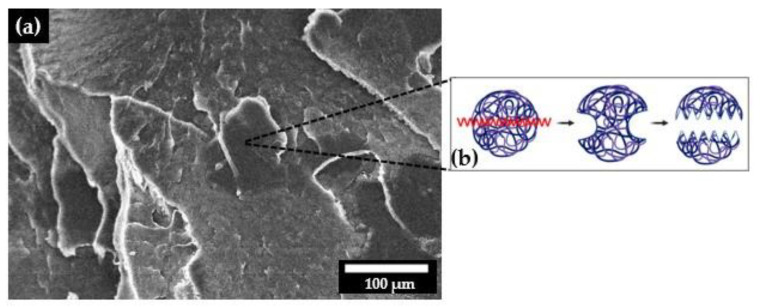
(**a**) SEM micrograph of the fracture surface of the UHMWPE particles and (**b**) mechanism of fracture of these particles, adapted from [36].

Another observed phenomenon related to the fracture surface involves cracks going through the UHMWPE particles and not around the interface between LLDPE and UHMWPE, probably indicating good interfacial adhesion (Figure 9a). This fracture behavior can be explained in terms of a mechanism called “bridge-breaking”, in which the dispersed particles are plastically deformed and then torn (Figure 9b) [37]. Furthermore, according to Boscoletto et al. [36], UHMWPE can be solubilized in the HDPE matrix by mixing in the solid state. They identified the same phenomenon for the HDPE/UHMWPE system. This dissolution may be responsible for the good interface, which is vital for good impact strength and the other mechanical properties of this LLDPE/UHMWPE system. Figure 9 shows the cryogenic fracture surface of some of the UHMWPE particles, proving the mechanism proposed in other studies [25,36]. Analyzing the micrograph (Figure 9a), one can observe a structure with grain boundaries that may be related to unmelted UHMWPE particles. Another striking feature is the irregular shape and different particle sizes of the dispersed phase. 

## 4. Conclusions

The addition of UHMWPE in LLDPE indicates, through thermal characterization by DSC, that a mixture with co-crystallization did not occur. This feature may come from the type of process used (solid state), which reduced the ability to form a homogeneous mixture between the polymers, maintaining the phases of each constituent as observed in the morphological analysis. 

The mechanical properties of the blends are in agreement with the thermal characteristics and the identified morphology, wherein tensile strength, elongation, and modulus of elasticity decreased with increasing UHMWPE content, due to the decrease in the degree of crystallinity.

The morphologies of the blends fail by cracking. The separation of the two phases has been identified, wherein the UHMWPE exhibits a dense configuration and a good interface without cracks, leading to efficient adhesion with the matrix.

## Figures and Tables

**Table 1:** Thermal behavior data of LLDPE, UHMWPE, and blends for first heating and cooling.

Formulation	1st Heating			Cooling		X_c_ (%)
	T_m1_ (°C)	T_m2_ (°C)	ΔH (J/g)	T_c1_ (°C)	T_c2_ (°C)	
LLDPE	127.0 ± 0.4	-----	153.1 ± 0.4	114.1 ± 0.1	-----	52.7 ± 0.1
15 UHMWPE	126.6 ± 0.4	132.4 ± 0.1	149.5 ± 0.1	113.9 ± 0.1	118.9 ± 0.2	46.0 ± 0.2
30 UHMWPE	126.8 ± 0.1	131.6 ± 0.2	159.5 ± 0.7	113.7 ± 0.1	118.8 ± 0.1	51.3 ± 0.1
45 UHMWPE	127.1 ± 0.9	131.5 ± 0.1	154.8 ± 0.1	113.5 ± 0.3	118.4 ± 0.2	53.0 ± 0.2
UHMWPE	-----------	132.7 ± 0.9	133.7 ± 0.4	----------	118.3 ± 0.1	45.3 ± 0.1

**Table 2:** Thermal behavior data of LLDPE, UHMWPE, and the blends for the second heating.

Formulation	2nd Heating			X_c_ (%)
	T_m1_ (°C)	T_m2_ (°C)	ΔH (J/g)	
LLDPE	127.2 ± 0.1	-----	154.4 ± 0.2	52.9 ± 0.1
15 UHMWPE	126.9 ± 0.2	132.3 ± 0.1	156.3 ± 0.1	53.5 ± 0.0
30 UHMWPE	127.1 ± 0.1	131.8 ± 0.1	164.3 ± 0.1	56.3 ± 0.1
45 UHMWPE	126.8 ± 0.1	132.3 ± 0.1	167.8 ± 0.1	57.5 ± 0.1
UHMWPE	---------	132.8 ± 0.1	153.7 ± 0.9	52.6 ± 0.2

**Table 3:** Tensile test data of LLDPE, UHMWPE, and blends.

Formulation	σr (MPa)	Ɛr (%)	E (MPa)
LLDPE	12.7 ± 1.1	175 ± 2	744 ± 9
15 UHMWPE	17.2 ± 0.7	359 ± 1	561 ± 7
30 UHMWPE	17.3 ± 0.5	328 ± 10	507 ± 58
45 UHMWPE	16.3 ± 3.8	210 ± 5	526 ± 7
UHMWPE	25.1 ± 0.7	187 ± 12	485 ± 12

## Data Availability

Not applicable.
